# Identifying when racial and ethnic disparities arise along the continuum of transplant care: a national registry study

**DOI:** 10.1016/j.lana.2024.100895

**Published:** 2024-10-03

**Authors:** Maya N. Clark-Cutaia, Gayathri Menon, Yiting Li, Garyn T. Metoyer, Mary Grace Bowring, Byoungjun Kim, Babak J. Orandi, Stephen P. Wall, Melissa D. Hladek, Tanjala S. Purnell, Dorry L. Segev, Mara A. McAdams-DeMarco

**Affiliations:** a Hunter-Bellevue School of Nursing, Hunter College, City University of New York, New York, NY, USA; b Department of Medicine, New York University Grossman School of Medicine, New York, NY, USA; c Department of Surgery, New York University Grossman School of Medicine, New York, NY, USA; d Division of Transplantation, Department of Surgery, Johns Hopkins School of Medicine, Baltimore, MD, USA; e Department of Population Health, New York University Grossman School of Medicine, New York, NY, USA; f Ronald O. Perelman Department of Emergency Medicine, New York University Grossman School of Medicine, New York, NY, USA; g Johns Hopkins University School of Nursing, Baltimore, MD, USA; h Department of Epidemiology, Johns Hopkins Bloomberg School of Public Health, Baltimore, MD, USA; i Department of Health Behavior and Society, Johns Hopkins Bloomberg School of Public Health, Baltimore, MD, USA; j Johns Hopkins Center for Health Equity, Johns Hopkins University, Maryland Public Health, Baltimore, MD, USA

**Keywords:** Kidney transplantation, Disparities, Health equity, Underrepresented minorities

## Abstract

**Background:**

Fewer minoritized patients with end-stage kidney disease (ESKD) receive kidney transplantation (KT); efforts to mitigate disparities have thus far failed. Pinpointing the specific stage(s) within the transplant care continuum (being informed of KT options, joining the waiting list, to receiving KT) where disparities emerge among each minoritized population is pivotal for achieving equity. We therefore quantified racial and ethnic disparities across the KT care continuum.

**Methods:**

We conducted a retrospective cohort study (2015–2020), with follow-up through 12/10/2021. Patients with incident dialysis were identified using the US national registry data. The exposure was race and ethnicity (Asian, Black, Hispanic, and White). We used adjusted modified Poisson regression to quantify the adjusted prevalence ratio (aPR) of being informed of KT, and cause-specific hazards models to calculate adjusted hazard ratios (aHR) of listing, and transplantation after listing.

**Findings:**

Among 637,951 adults initiating dialysis, the mean age (SD) was 63.8 (14.6), 41.8% were female, 5.4% were Asian, 26.3% were Black, 16.6% were Hispanic, and 51.7% were White (median follow-up in years [IQR]:1.92 [0.97–3.39]). Black and Hispanic patients were modestly more likely to be informed of KT (Black: aPR = 1.02, 95% confidence interval [CI]:1.01–1.02; Hispanic: aPR = 1.03, 95% CI: 1.02–1.03) relative to White patients. Asian patients were more likely to be listed (aHR = 1.18, 95% CI: 1.15–1.21) but less likely to receive KT (aHR = 0.56, 95% CI: 0.54–0.58). Both Black and Hispanic patients were less likely to be listed (Black: aHR = 0.87, 95% CI: 0.85–0.88; Hispanic: aHR = 0.85, 95% CI: 0.85–0.88) and receive KT (Black: aHR = 0.61, 95% CI: 0.60–0.63; Hispanic: aHR = 0.64, 95% CI: 0.63–0.66).

**Interpretation:**

Improved characterization of the barriers in KT access specific to each racial and ethnic group, and the interventions to address these distinct challenges throughout the KT care continuum are needed; our findings identify specific stages most in need of mitigation.

**Funding:**

10.13039/100000002National Institutes of Health.

**Research in context**

**Evidence before this study**

We conducted a literature search on March 15, 2023, using both PubMed and Google Scholar with the following search terms: “informed of kidney transplantation”, “access to waitlisting”, “race and ethnicity and access to waitlisting”, “kidney transplantation”, “access to kidney transplantation”, “race and ethnicity and access to kidney transplantation”, “race and ethnicity and access to live-donor kidney transplantation”, “race and ethnicity and access to deceased-donor kidney transplantation”, “race and ethnicity and access to preemptive kidney transplantation”, and “Kidney Allocation System”. We found that many studies are single-center or regional studies that characterized racial and ethnic disparities in the earlier steps of the kidney transplant care continuum. Additionally, national studies have only examined candidates on the waitlist, or transplant recipients. However, we did not identify any national-scale studies examining access to kidney transplantation from the initiation of dialysis to transplantation, especially after the implementation of Kidney Allocation System. Furthermore, existing studies have primarily focused on disparities between Black and White individuals, with fewer examining the challenges faced by Asian and Hispanic individuals.

**Added value of this study**

This study quantified the magnitude of and trends in racial and ethnic (non-Hispanic Asian, non-Hispanic Black, Hispanic and non-Hispanic White) disparities in being informed of, listing for, and receiving kidney transplantation after the implementation of the Kidney Allocation System (2015–2020). We found that a higher proportion of Black and Hispanic patients with end-stage kidney disease were informed of kidney transplantation, but they had a lower likelihood of being listed (Black = 12% lower, Hispanic = 14% lower) and obtaining kidney transplantation (Black = 38% lower, Hispanic = 36% lower) relative to White patients. Asian patients with end-stage kidney disease were 21% more likely to be listed but 45% less likely to obtain kidney transplantation after listing.

**Implications of all the available evidence**

We found that minoritized patients (non-Hispanic Asian, non-Hispanic Black, and Hispanic) had a modestly higher likelihood of being informed of kidney transplantation than White patients after the implementation of the Kidney Allocation System; however, they continued to face significant challenges in accessing kidney transplantation thereafter. This disparity likely stems from a complex interplay of systemic and individual factors acting as barriers across key steps of the kidney transplant care continuum. Our findings highlight the pressing need for targeted interventions tailored to the unique barriers faced by each racial and ethnic group across the kidney transplant care continuum.

## Introduction

Kidney transplantation is the preferred treatment for end-stage kidney disease, offering improved survival and quality of life.^1^ Substantial efforts have been made over the past two decades to reduce racial and ethnic disparities across the kidney transplant care continuum.^2^, ^3^, ^4^, ^5^, ^6^, ^7^, ^8^, ^9^, ^10^, ^11^, ^12^ These efforts ranged from addressing knowledge gaps surrounding live donor kidney transplantation, which is associated with superior outcomes,^3^^,^^4^^,^^13^ to the implementation of the Kidney Allocation System in 2014 to reduce wait times to deceased donor kidney transplantation for listed candidates.^5^, ^6^, ^7^, ^8^, ^9^ However, early evidence suggests these efforts have not significantly mitigated racial and ethnic disparities.^4^^,^^14^, ^15^, ^16^ For instance, in the first few years after the Kidney Allocation System, access to kidney transplantation and preemptive kidney transplantation were lower among racial and ethnic minorities relative to White individuals.^15^^,^^17^

The process of obtaining kidney transplantation involves several crucial steps, from end-stage kidney disease diagnosis to receiving education about kidney transplantation, referral, evaluation, listing, and ultimately transplantation. However, the steps of transplant are not necessarily linear-where kidney transplant education may be received much earlier than end-stage kidney disease diagnosis. Single-center and regional studies have characterized racial and ethnic disparities in the earlier stages of the kidney transplant care continuum,^16^^,^^18^^,^^19^ but most national studies examining such disparities tend to focus on waitlisted patients or transplant recipients.^4^^,^^14^^,^^15^^,^^17^^,^^20^, ^21^, ^22^, ^23^, ^24^, ^25^, ^26^, ^27^, ^28^ The specific stages along the kidney transplant care continuum-specifically, being informed of kidney transplantation at dialysis initiation, listing, and transplantation-where disparities arise for minoritized groups are not well characterized. To the best of our knowledge, there have been no national studies to date that have quantified racial and ethnic disparities in access to transplantation over the kidney transplant care continuum, starting from the initiation of dialysis to transplantation.

Furthermore, while a majority of existing studies have primarily focused on disparities between Black and White individuals,^16^^,^^18^^,^^22^, ^23^, ^24^, ^25^^,^^27^, ^28^, ^29^, ^30^, ^31^, ^32^, ^33^, ^34^, ^35^, ^36^ fewer have highlighted the challenges faced by Asian and Hispanic individuals,^4^^,^^14^^,^^19^^,^^37^, ^38^, ^39^, ^40^, ^41^, ^42^ particularly following the implementation of the Kidney Allocation System. Considering the growing Asian and Hispanic population with end-stage kidney disease population in the United States,^43^ it is critical to understand if and when these groups encounter disparities along the kidney transplant care continuum. While single-center studies are crucial in recognizing the mechanisms contributing to and perpetuating disparities in the earlier steps of the kidney transplant care continuum, they need to be contextualized in the broader national landscape of transplantation to guide targeted interventions aimed at fostering greater equity in transplantation. Moreover, pinpointing intervention strategies tailored to each racial and ethnic group is pivotal for optimizing resource allocation and improving transplantation outcomes on a wider scale.

Therefore, we estimated the likelihood of being informed of kidney transplantation and quantified the magnitude of and trends in disparities in access to kidney transplant waitlisting following initiation of dialysis, and first kidney transplantation (any kidney transplantation, deceased donor kidney transplantation, live donor kidney transplantation, and preemptive kidney transplantation) after listing for Asian, Black, Hispanic, and White adults after the implementation of Kidney Allocation System.

## Methods

This study was reviewed by the Institutional Review Board at the New York University Grossman School of Medicine and was determined to qualify for an exemption under s22-0053, waiving the need for informed consent as patients could not be identified. This study followed the Strengthening the Reporting of Observational Studies in Epidemiology (STROBE) guidelines for cohort studies.^44^

### Study design and participants

**Fig. 1:**
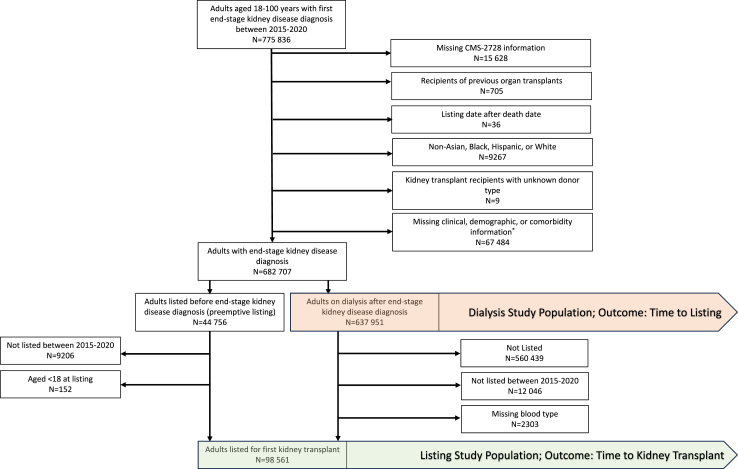
**Cohort development.** ∗353 individuals with missing comorbidities, 66980 with missing body mass index, and 647 with missing cause of end-stage kidney disease.

We studied adults (aged ≥18) with end-stage kidney disease in the United States between January 1, 2015 and December 31, 2020. This study comprises two analytic populations: adults with end-stage kidney disease initiating dialysis (to quantify access to listing) and adults listed for a kidney transplant (to quantify access to transplantation among listed candidates) (Fig. 1, Supplementary Figure S1). The dialysis population included 637,951 adults (age ≥18 years) who initiated dialysis between January 1, 2015 and December 31, 2020, and the listing population consisted of 98,561 adults from the dialysis population who were listed between January 1, 2015 and December 31, 2020. The listing population also included adults with end-stage kidney disease who were listed before initiating dialysis between January 1, 2015 and December 31, 2020 (N = 35,398). This timeframe allows us to identify trends in access to kidney transplantation after the implementation of Kidney Allocation System in December 2014. In both populations, race and ethnicity were designated one of the following categories: non-Hispanic Asian (Asian American, Native Hawaiian and Pacific Islander; Asian hereafter), non-Hispanic Black, Hispanic/Latino (Hispanic hereafter), or non-Hispanic White (White hereafter) (see exposure section).

### Data source

This study used data from the United States Renal Data System (USRDS). The USRDS database serves as a comprehensive national registry, capturing data on patients with kidney failure through a collaborative effort involving the Center for Medicare & Medicaid Services (CMS), the United Network for Organ Sharing, and the end-stage kidney disease Networks.^43^ We obtained information related to patients’ knowledge of their kidney transplant options at dialysis initiation, comorbidities, employment status, and body mass index (BMI) from the Centers for Medicare & Medicaid Services (CMS) Medical Evidence Report (CMS-2728),^45^^,^^46^ as well as the patient and waitlist datasets provided by CMS and Organ Procurement and Transplantation Network (OPTN). Unless stated otherwise, covariates were measured at the initiation of dialysis.

### Exposure: race and ethnicity

Provider-reported race and ethnicity information were obtained from the USRDS patient files,^4^ which codes race (White, Black, American Indian/Alaska Native, Asian, Native Hawaiian/other Pacific Islander, Mid-East/Arabian, Indian subcontinent, or Unknown) and ethnicity (Hispanic and non-Hispanic) into separate categories.^46^ We used both the race and ethnicity variables provided by the USRDS to categorize individuals into the aforementioned racial and ethnic categories. To quantify disparities, White race was used as the reference category.

### Covariates: participant characteristics

Based on the National Institute on Minority Health and Health Disparities Research Framework and previously published literature,^47^^,^^48^ we considered the following characteristics as potential covariates: age (in years at dialysis initiation, listing; categories: 18–34, 35–49, 50–64, ≥65), the year of dialysis/listing (2015–2016, 2017–2018, and 2019–2020), sex, body mass index (BMI in kg/m^2^; categories: 14–17.9, 18–24.9, 25–29.9, ≥30; BMI at listing was used for preemptively listed candidates), Medicare coverage (at dialysis), cause of end-stage kidney disease, comorbidities (hypertension, diabetes, heart failure, atherosclerotic heart disease, peripheral vascular disease, cerebrovascular disease, chronic obstructive pulmonary disease, cancer, functional impairment, inability to transfer, alcohol use, tobacco use), blood group (for access to transplantation only), and calculated panel reactive antibody (for access to transplantation only; categories: <80%, and ≥80%).

### Outcomes: access to kidney transplant listing and first kidney transplantation

The outcomes of interest were: the likelihood of listing after the dialysis initiation (dialysis population) and the likelihood of kidney transplantation (any kidney transplantation, deceased donor kidney transplantation, live donor kidney transplantation, and preemptive kidney transplantation) after listing (listing population). Preemptive kidney transplantation is defined as kidney transplantation without prior history of dialysis. Outcomes were ascertained using dates of listing/transplantation in the national registry.

### Statistical analysis

The analyses presented are based on a complete case analysis (Fig. 1). The distributions of transplant candidate characteristics were summarized with the mean and standard deviation (SD), median and interquartile range (IQR) for continuous variables, and with N (%) for categorical variables.

#### Provision of kidney transplant information at dialysis initiation

We determined the crude proportions of patients informed of kidney transplantation at dialysis initiation by race and ethnicity. We then used modified Poisson regression models,^49^ adjusting for clinical and demographic factors, to quantify the adjusted prevalence ratio (aPR) of patients being informed of kidney transplantation by race and ethnicity.

#### Access to listing- dialysis population

We utilized the Kaplan–Meier method to estimate the unadjusted cumulative incidence of listing after initiation of dialysis, stratified by patient race and ethnicity. The P-values for Kaplan–Meier plots were obtained using the log-rank test. Patients were followed from the date of first dialysis to the date of listing. All outcomes were censored at the first date on which any of the following events occurred: listing, kidney transplant, death, or end of study observation period (December 10, 2021). Cause-specific hazards models were used to estimate the association between race and ethnicity and the time to first listing.

#### Access to transplantation- listing population

We utilized the Kaplan–Meier method to estimate the unadjusted cumulative incidence of first kidney transplant after listing (any kidney transplant, deceased donor kidney transplant, live donor kidney transplant, and preemptive kidney transplant), stratified by race and ethnicity. We followed the listed candidates from the date of listing to the date of first kidney transplant. All outcomes were censored on the first date on which the following events occurred: kidney transplant, death, removal from the waitlist, or December 10, 2021. Waitlist removal could be due to (amongst other reasons)- death, kidney transplant, refusal of kidney transplant, improvement in condition, transplant in another country, and unable to contact candidate. For a particular type of kidney transplant, obtaining a different type of kidney transplant was treated as a censoring event. Cause-specific hazards models were used to estimate the association between race and ethnicity and time to first kidney transplant (any kidney transplant, deceased donor kidney transplant, live donor kidney transplant, and preemptive kidney transplant) among candidates.

#### Secondary analyses

To examine trends in access to listing and transplantation, respectively, we utilized an interaction term between candidate race and ethnicity and years of dialysis initiation and listing, respectively. We used the Wald test to determine the P-values of the interaction terms.

We further quantified the reasons for which patients with end-stage kidney disease were reported to be not informed of kidney transplantation, overall and by individual race and ethnicity. Finally, we quantified access to listing and transplantation by race and ethnicity and informed status using cause-specific hazards models with an interaction between informed status and race and ethnicity, adjusted for the aforementioned variables.

#### Sensitivity analysis

We tested the robustness of our estimates through: 1) Fine and Gray sub-distribution hazards models (competing risks for access to listing: death and kidney transplant; competing risk for access to kidney transplantation: death); 2) limiting our study populations to individuals with Medicare at the time of dialysis initiation; 3) time-varying cause-specific hazards models, allowing for the interaction between race and ethnicity and time model for changes in hazard ratios over time; 4) creating separate categories for missing values of variables^4^; 5) including preemptively listed individuals (individuals listed before dialysis initiation) into the dialysis population; 6) including employment as a covariate in cause-specific hazards models; 7) analyzing Asian American and Native Hawaiian/Pacific Islander individuals separately; and 8) utilizing the composite outcome of listing and kidney transplantation in the dialysis population.

All statistical analyses were conducted using SAS (v9.4) and Stata (v17, Stata Corp, College Station, TX). Statistical significance was defined as a two-sided P value < 0.05.

### Role of funding source

The funder of the study had no role in study design, data collection, data analysis, data interpretation, or writing of the manuscript.

## Results

### Dialysis study population

The dialysis study population (N = 637,951) was 16.6% Hispanic, 5.4% Asian, 26.3% Black, and 51.7% White; the mean age was 63.8 years (SD = 14.6). Among dialysis patients, 41.8% were female, 49.0% had diabetes mellitus, and 30.4% had hypertension as the cause of end-stage kidney disease (Table 1).

**Table 1:** Characteristics of the adults diagnosed with end-stage kidney disease who initiated dialysis (2015–2020).

	Total	Asian^a^	Black	Hispanic	White
	N = 637,951	N = 34,639	N = 167,687	N = 106,060	N = 329,565
**Age in years, mean (SD)**^b^	63.8 (14.6)	64.3 (15.1)	60.4 (14.6)	59.7 (14.9)	66.8 (13.7)
**Age in years, No. (%)**^b^					
18–34	26,770 (4.2)	1526 (4.4)	9719 (5.8)	6748 (6.4)	8777 (2.7)
35–49	78,475 (12.3)	4272 (12.3)	28,028 (16.7)	18,508 (17.5)	27,667 (8.4)
50–64	198,355 (31.1)	10,098 (29.2)	59,280 (35.4)	38,472 (36.3)	90,505 (27.5)
≥65	334,351 (52.4)	18,743 (54.1)	70,660 (42.1)	42,332 (39.9)	202,616 (61.5)
**Female, No. (%)**	266,636 (41.8)	14,535 (42.0)	78,127 (46.6)	42,562 (40.1)	131,412 (39.9)
**Primary cause of end-stage kidney disease, No. (%)**					
Diabetes	312,547 (49.0)	19,481 (56.2)	73,834 (44.0)	65,739 (62.0)	153,493 (46.6)
Hypertension	194,226 (30.4)	9135 (26.4)	67,713 (40.4)	24,218 (22.8)	93,160 (28.3)
Glomerulonephritis	39,535 (6.2)	2792 (8.1)	8778 (5.2)	6180 (5.8)	21,785 (6.6)
Others	91,643 (14.4)	3231 (9.3)	17,362 (10.4)	9923 (9.4)	61,127 (18.5)
**BMI in kg/m**^**2**^**, median[IQR]**	28.2 [24.0–33.7]	25.1 [22.0–29.3]	28.5 [24.1–34.4]	27.6 [24.0–32.4]	28.6 [24.2–34.2]
**BMI in kg/m**^**2**^**, No. (%)**					
14–17.9	14,251 (2.2)	1394 (4.0)	4236 (2.5)	1779 (1.7)	6842 (2.1)
18–24.9	184,226 (28.9)	15,812 (45.6)	46,612 (27.8)	32,019 (30.2)	89,783 (27.2)
25–29.9	180,886 (28.4)	9778 (28.2)	44,962 (26.8)	34,122 (32.2)	92,024 (27.9)
≥30	258,588 (40.5)	7655 (22.1)	71,877 (42.9)	38,140 (36.0)	140,916 (42.8)
**Comorbidities, No. (%)**^b^					
Diabetes	386,290 (60.6)	22,233 (64.2)	99,008 (59.0)	72,848 (68.7)	192,201 (58.3)
Hypertension	561,631 (88.0)	30,926 (89.3)	153,029 (91.3)	94,498 (89.1)	283,178 (85.9)
Alcohol dependence	11,126 (1.7)	205 (0.6)	3239 (1.9)	1586 (1.5)	6096 (1.8)
Cancer	46,016 (7.2)	1382 (4.0)	9447 (5.6)	3829 (3.6)	31,358 (9.5)
Atherosclerotic heart disease	85,708 (13.4)	4025 (11.6)	15,382 (9.2)	11,706 (11.0)	54,595 (16.6)
Cerebrovascular disease	56,969 (8.9)	2616 (7.6)	17,440 (10.4)	7062 (6.7)	29,851 (9.1)
Heart failure	189,712 (29.7)	8057 (23.3)	49,495 (29.5)	24,802 (23.4)	107,358 (32.6)
Inability to transfer	24,800 (3.9)	1085 (3.1)	6852 (4.1)	3626 (3.4)	13,237 (4.0)
Tobacco use	43,105 (6.8)	973 (2.8)	13,084 (7.8)	3150 (3.0)	25,898 (7.9)
Peripheral vascular disease	62,303 (9.8)	1955 (5.6)	12,646 (7.5)	10,144 (9.6)	37,558 (11.4)
Chronic obstructive pulmonary disease	60,968 (9.6)	1230 (3.6)	12,905 (7.7)	4141 (3.9)	42,692 (13.0)
Functional impairment	102,435 (16.1)	4799 (13.9)	25,072 (15.0)	16,066 (15.1)	56,498 (17.1)
**Medicare, No. (%)**^b^	402,212 (63.0)	18,060 (52.1)	96,419 (57.5)	49,860 (47.0)	237,873 (72.2)
**Employment Status, No. (%)**^b^					
Employed	66,215 (10.4)	4951 (14.3)	19,323 (11.5)	11,514 (10.9)	30,427 (9.2)
Unemployed	157,413 (24.7)	9565 (27.6)	51,573 (30.8)	38,548 (36.3)	57,727 (17.5)
Retired	385,429 (60.4)	17,829 (51.5)	89,747 (53.5)	49,083 (46.3)	228,770 (69.4)
Other	28,894 (4.5)	2294 (6.6)	7044 (4.2)	6915 (6.5)	12,641 (3.8)
**Nephrology care prior to end-stage kidney disease diagnosis, No. (%)**^b^					
Yes	407,821 (63.9)	22,740 (65.6)	99,829 (59.5)	59,707 (56.3)	225,545 (68.4)
No	130,823 (20.5)	6603 (19.1)	36,932 (22.0)	28,404 (26.8)	58,884 (17.9)
Unknown	99,307 (15.6)	5296 (15.3)	30,926 (18.4)	17,949 (16.9)	45,136 (13.7)

SD, standard deviation; BMI, body mass index; IQR, interquartile range; PRA, panel-reactive antibody.

a Asian includes Asian American, Native Hawaiian and Pacific Islanders.

b At end-stage kidney disease diagnosis.

### Provision of Kidney Transplant Information at dialysis initiation

From 2015 to 2020, a higher proportion of Asian (85.4%), Black (87.5%), and Hispanic (88.7%) patients were informed of kidney transplantation than White patients (84.3%) (Supplementary Table S1). After adjustment, Black and Hispanic patients had a slightly higher prevalence of being informed of kidney transplantation relative to White patients, which was not observed among Asian patients: Asian: aPR = 1.00 (95% confidence interval [CI]):0.99–1.00), Black: aPR = 1.02 (95% CI: 1.01–1.02), Hispanic: aPR = 1.03 (95% CI: 1.02–1.03) (Table 2).

**Table 2:** Informed of kidney transplantation by race and ethnicity among adults diagnosed with end-stage kidney disease (2015–2020) (N = 669,769).

	Prevalence ratio (PR) (95% Confidence Interval)			
	White^a^	Asian^a^	Black^a^	Hispanic
Crude	Reference	**1.01 (1.01**–**1.02)**	**1.04 (1.03**–**1.04)**	**1.05 (1.04**–**1.05)**
Adjusted^b^	Reference	1.00 (0.99–1.00)	**1.02 (1.01**–**1.02)**	**1.03 (1.02**–**1.03)**

Bold denotes statistically significant at a two-sided P-value of 0.05. Individuals missing information status were excluded from this analysis (N = 12,938 excluded).

a Non-Hispanic White, Black and Asian; Asian includes Asian American, Native Hawaiian, and Pacific Islander.

b Adjusted PR of being informed of kidney transplantation at dialysis initiation (adjusted for: year of dialysis initiation, cause of end-stage kidney disease [diabetes, hypertension, glomerulonephritis, and other], sex, age at dialysis, body mass index, comorbidities [hypertension, diabetes, heart failure, atherosclerotic heart disease, peripheral vascular disease, cerebrovascular disease, chronic obstructive pulmonary disease, cancer, functional impairment, inability to transfer, alcohol use, tobacco use], and Medicare at dialysis initiation). Modified Poisson regression with robust variance estimator.

### Racial and ethnic disparities in access to first listing

**Fig. 2:**
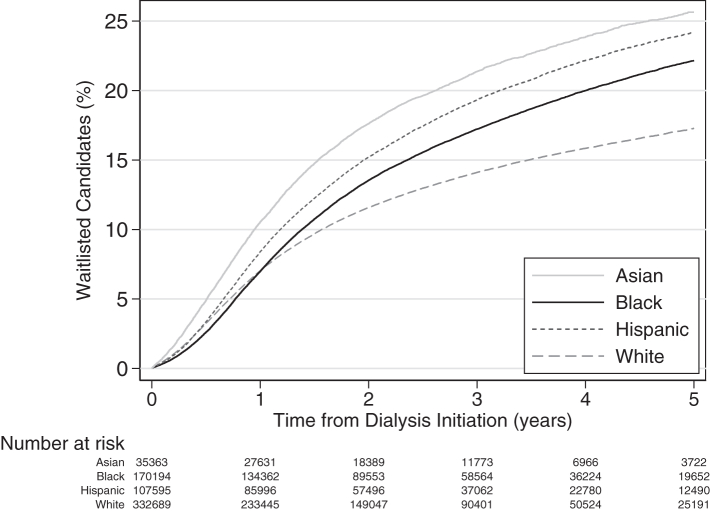
**Access to listing for Asian, Black, and Hispanic adults (age ≥18 years) with end-stage kidney disease after the initiation of dialysis, compared to White adults with end-stage kidney disease (2015**–**2020).** Kaplan–Meier curves depicting the unadjusted cumulative incidence of incident listing after dialysis initiation, excluding individuals who were listed prior to their diagnosis of end-stage kidney disease.

The 5-year unadjusted cumulative incidence of listing after dialysis initiation was higher among Asian (23.6%, 95% CI: 23.0–24.2), Black (20.5%, 95% CI: 20.2–20.8), and Hispanic (22.8%, 95% CI: 23.4–23.1) patients compared to White patients (16.1%, 95% CI: 15.9–16.3) (*P*_*Log-rank*_<0.0001) (Fig. 2; Supplementary Table S2). Similarly, the incidence rate of listing (per 1000 person-years) was higher among Asian (69.0, 95% CI: 67.3–70.8), Black (53.7, 95% CI: 53.0–54.1), and Hispanic (61.9, 95% CI: 60.9–62.8) patients compared to White patients (46.2, 95% CI: 45.7–46.7) (median follow-up in years [interquartile range]: 1.8 years [0.7–3.3]; total follow-up: 6.9 years).

After adjustment, Asian patients had a 18% higher likelihood of listing (adjusted hazard ratio (aHR) = 1.18, 95% CI: 1.15–1.21), compared to White patients. However, Black and Hispanic patients had a lower likelihood of listing (Black: aHR = 0.87, 95% CI: 0.85–0.88; Hispanic: aHR = 0.86, 95% CI: 0.85–0.88) (Table 3).

**Table 3:** Access to key stages on the kidney transplant care continuum by race and ethnicity (2015–2020).

	Adjusted hazard ratio (aHR) (95% Confidence Interval)^a^			
	White^b^	Asian^b^	Black^b^	Hispanic
**Listed** (N = 637,951)	Reference	**1.18 (1.15**–**1.21)**	**0.87 (0.85**–**0.88)**	**0.86 (0.85**–**0.88)**
Cases	32,354	5855	22,486	16,520
**Transplanted** (N = 98,561)^c^				
**Any kidney transplant**	Reference	**0.56 (0.54**–**0.58)**	**0.61 (0.60**–**0.63)**	**0.64 (0.63**–**0.66)**
Cases	27,132	3064	9418	7337
Deceased donor kidney transplant	Reference	**0.69 (0.66**–**0.73)**	**0.92 (0.89**–**0.94)**	**0.79 (0.76**–**0.82)**
Cases	11,604	1776	6734	4119
Live donor kidney transplant	Reference	**0.43 (0.40**–**0.47)**	**0.34 (0.33**–**0.36)**	**0.55 (0.53**–**0.58)**
Cases	8329	800	1729	2192
Preemptive kidney transplant	Reference	**0.46 (0.42**–**0.51)**	**0.35 (0.32**–**0.37)**	**0.47 (0.44**–**0.51)**
Cases	7199	488	955	1026

Bold denotes statistically significant at a two-sided P-value of 0.05.

a Adjusted HR of being listed in the dialysis population (adjusted for: year of dialysis initiation, cause of end-stage kidney disease (diabetes, hypertension, glomerulonephritis, and other), sex, age at dialysis initiation, body mass index (BMI), comorbidities [hypertension, diabetes, heart failure, atherosclerotic heart disease, peripheral vascular disease, cerebrovascular disease, chronic obstructive pulmonary disease, cancer, functional impairment, inability to transfer, alcohol use, tobacco use], and Medicare at dialysis initiation). Adjusted HR of being transplanted after listing (adjusted for: year of listing, cause of end-stage kidney disease, sex, age at listing, BMI, comorbidities, blood group, calculated panel reactive antibody, and Medicare at dialysis initiation).

b Non-Hispanic White, Black and Asian; Asian includes Asian American, Native Hawaiian, and Pacific Islander.

c Includes individuals who were listed prior to dialysis initiation.

Over time, the likelihood of listing decreased for minoritized patients relative to White patients (Supplementary Table S3): Black (aHR = 0.80, 95% CI: 0.77–0.82) and Hispanic patients (aHR = 0.72, 95% CI: 0.69–0.75) were less likely to be listed in 2019–2020. However, Asian patients had a comparable likelihood of listing relative to White patients in 2019–2020 (aHR = 1.05, 95% CI: 0.99–1.10).

### Listing study population

The listing study population (N = 98,561) was 7.7% Asian, 18.7% Hispanic, 24.7% Black, and 48.9% White; the mean age at listing was 53.4 (SD = 13.2) years, 36.9% were female, 36.6% had diabetes mellitus, and 26.3% had hypertension as the cause of end-stage kidney disease (Supplementary Table S4).

### Racial and ethnic disparities in access to first kidney transplantation

**Fig. 3:**
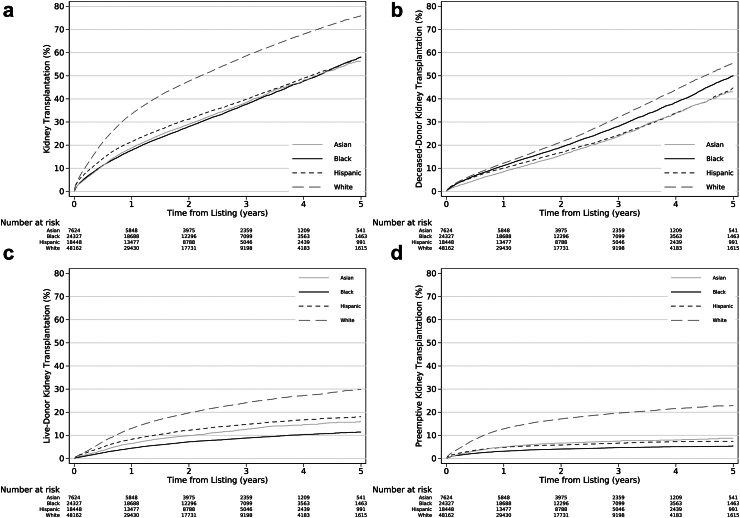
**Access to (a) any kidney transplant, (b) deceased donor kidney transplant, (c) live donor transplant, and (d) preemptive kidney transplant among candidates on the waitlist by candidate race and ethnicity (2015**–**2020).** Kaplan–Meier curves depicting the unadjusted cumulative incidence of kidney transplantation after listing. This population includes candidates on the waitlist who were listed prior to their diagnosis of end-stage kidney disease.

The 5-year cumulative incidence of kidney transplantation among White waitlisted candidates (75.9%, 95% CI: 75.3–76.6) was significantly higher than that of Asian (56.4%, 95% CI: 54.6–58.1), Black (58.1%, 95% CI: 57.1–59.2), and Hispanic (58.1%, 95% CI: 56.9–59.4) candidates (*P*_*Log-rank*_<0.0001; Fig. 3, Supplementary Table S2). Similarly, the incidence rate of kidney transplantation (per 1000 person-years) was higher among White candidates than minoritized candidates (White: 323.5, 95% CI: 319.7–327.4; Asian: 173.6, 95% CI: 167.6–179.9; Black: 172.1, 95% CI: 168.7–175.6; Hispanic: 186.5, 95% CI: 182.3–190.8). After adjustment, Asian (aHR = 0.56, 95% CI: 0.54–0.58), Black (aHR = 0.61, 95% CI: 0.60–0.63), and Hispanic candidates (aHR = 0.64, 95% CI: 0.63–0.66) had a significantly lower likelihood of receiving any kidney transplant relative to White candidates (Table 3) (median follow-up in years [interquartile range]: 1.7 years [0.7–2.9]; total follow-up: 6.9 years).

The largest disparities among candidates were for live donor kidney transplantation (Fig. 3, Supplementary Table S2): the 5-year unadjusted cumulative incidence of live donor kidney transplantation for White candidates (29.9%, 95% CI: 29.1–30.7) was higher than that for Asian (16.0%, 95% CI: 14.7–17.3), Black (11.4%, 95% CI: 10.8–12.1), and Hispanic (18.1%, 95% CI: 17.2–19.0) candidates (*P*_*Log-rank*_ <0.0001) (Fig. 3, Supplementary Table S2).

After adjustment, Asian (aHR = 0.69, 95% CI: 0.66–0.73), Black (aHR = 0.92, 95% CI: 0.89–0.94), and Hispanic (aHR = 0.79, 95% CI: 0.76–0.82) candidates had a significantly lower likelihood of deceased donor kidney transplantation compared to White candidates (Table 3). Asian (aHR = 0.43, 95% CI: 0.40–0.47), Black (aHR = 0.34, 95% CI: 0.33–0.36), and Hispanic (aHR = 0.55, 95% CI: 0.53–0.58) candidates had a significantly lower likelihood of live donor kidney transplantation relative to White candidates. Similar associations were observed among minoritized candidates’ access to preemptive kidney transplantation (Table 3).

The likelihood of any kidney transplantation, deceased donor kidney transplantation, live donor kidney transplantation, and preemptive kidney transplantation among minoritized candidates remained below that for White candidates during all eras (Supplementary Table S5). Over time, there was a significant decrease in access to deceased donor kidney transplantation for Asian candidates relative to White candidates. Furthermore, access to live donor kidney transplantation and preemptive kidney transplantation decreased significantly for Black and Hispanic candidates, but remained unchanged for Asian candidates relative to White candidates (Supplementary Table S5).

### Access to listing and kidney transplantation by informed status

Among non-informed patients, a higher proportion of Asian (22.1%) and White patients (21.0%) were not informed due to unsuitable age, compared to Black (13.8%) and Hispanic patients (14.9%). A higher proportion of Black (59.9%) and Hispanic (60.8%) patients were reported to be non-informed due to being unassessed, relative to Asian (44.3%) and White non-informed patients (49.7%; Supplementary Table S6). The likelihood of listing did not differ by racial and ethnic groups among non-informed patients relative to White patients (Asian: aHR = 1.01, 95% CI: 0.90–1.03; Black: aHR = 1.06, 95% CI: 0.99–1.14; Hispanic: aHR = 1.00, 95% CI: 0.93–1.08). However, among informed patients, Asian patients were more likely to be listed (aHR = 1.21, 95% CI: 1.17–1.24; P_interaction_ = 0.002), while Black (aHR = 0.85, 95% CI: 0.84–0.87; P_interaction_<0.0001) and Hispanic patients (aHR = 0.85, 95% CI: 0.84–0.87; P_interaction_<0.0001) were significantly less like to be listed than their White counterparts. After listing, however, minoritized candidates were less likely to obtain kidney transplantation than their White counterparts, with no difference by information status (all P_interaction_>0.05) (Supplemental Table S7).

### Sensitivity analysis

Our findings were robust to the following analyses: (1) Fine and Gray sub-distribution hazards models (Supplementary Table S8); (2) restricting study populations to Medicare beneficiaries (Supplementary Table S9); (3) time-varying cause-specific hazards models (Supplementary Table S10); and (4) adding separate categories for missing predictors (Supplementary Table S11); 5) including preemptively listed individuals to quantify access to listing (Supplementary Table S12); 6) including employment as a covariate in cause-specific hazards models (Supplementary Table S13); 7) analyzing Asian American and Native Hawaiian/Pacific Islander individuals separately (Supplementary Table S14); and 8) utilizing the composite outcome of listing and kidney transplantation (Supplementary Table S15).

## Discussion

In this national study of 637,951 patients with end-stage kidney disease and 98,561 transplant candidates, we identified the critical stages in the kidney transplant care continuum where racial and ethnic disparities arise in the post-Kidney Allocation System era. The prevalence of minoritized patients (Black and Hispanic) who were informed of kidney transplantation at dialysis initiation were modestly higher relative to White patients, yet disparities arose thereafter. Asian patients experienced an 18% higher likelihood of listing, but 44% lower access to kidney transplantation compared to their White counterparts; while Black and Hispanic patients had lower access to both listing (Black: 13%; Hispanic: 14%) and kidney transplantation (Black: 39%; Hispanic: 36%). Notably, all minoritized candidates had a strikingly lower likelihood of live donor kidney transplantation and preemptive kidney transplantation than their White counterparts. Lastly, being informed of kidney transplantation at dialysis initiation was associated with a higher likelihood of listing among Asian patients, and a significantly lower likelihood of listing among Black and Hispanic patients relative to White patients; however, after listing, minoritized patients had a lower likelihood of obtaining kidney transplantation relative to White candidates, regardless of their information status.

Our study expands upon previous research,^16^^,^^28^^,^^33^^,^^50^, ^51^, ^52^, ^53^, ^54^ highlighting persistent disparities in access to kidney transplantation using contemporary data, and identifying key steps across the kidney transplant care continuum where disparities are most prominent for each racial and ethnic minority group. Specifically, our findings reveal that while Asian patients were more likely to be listed for kidney transplantation, they were less likely to ultimately receive kidney transplant after the implementation of the Kidney Allocation System. Despite factors such as lower comorbidity rates and higher socioeconomic status in this population,^51^^,^^55^, ^56^, ^57^ which are typically associated with increased listing, disparities in access to kidney transplantation persisted among Asian adults after listing. This suggests that other non-clinical factors identified in literature may hinder kidney transplantation access, and lead to the observed associations: sociocultural beliefs, attitudes,^58^ inadequate linguistically- and culturally-tailored care,^59^, ^60^, ^61^, ^62^, ^63^, ^64^ and health literacy.^51^^,^^54^^,^^65^, ^66^, ^67^, ^68^, ^69^ Moreover, prior studies have also identified live donor kidney transplantation-specific challenges, including donor shortages,^70^ neighborhood poverty,^42^ and knowledge gaps among potential live donors and recipients. These factors likely impacted the associations observed and exacerbated extant disparities, particularly affecting access to live donor kidney transplantation and preemptive kidney transplantation among Asian adults post-Kidney Allocation System.^2^^,^^35^^,^^71^

Similarly, our findings reveal that Hispanic patients experienced a lower likelihood of listing and a lower likelihood of transplantation after listing than their White counterparts, consistent with prior literature.^4^^,^^17^^,^^19^^,^^37^^,^^39^^,^^40^^,^^72^ Similar to Asian adults, Hispanic adults have been documented in the literature to face challenges in accessing the waitlist and undergoing transplantation due to linguistic and sociocultural barriers.^2^^,^^73^^,^^74^ Moreover, they may encounter unique challenges in procuring living donors, attributable to both clinical and non-clinical factors.^2^^,^^18^^,^^35^^,^^71^^,^^75^ This suggests that there may be an overlap in the barriers to kidney transplantation faced by non-Black minoritized populations post-Kidney Allocation System, warranting further investigation to elucidate the challenges specific to each group.

Additionally, we found that Black patients experienced a lower likelihood of being listed and undergoing kidney transplantation across the care continuum relative to White candidates, despite the implementation of the Kidney Allocation System. Similar to findings in other minoritized groups, previous studies have identified factors such as reduced healthcare access,^76^ knowledge gaps,^75^^,^^77^^,^^78^ provider perceptions,^79^^,^^80^ socioeconomic status,^29^^,^^31^^,^^57^^,^^81^^,^^82^ and neighborhood characteristics that are associated with reduced likelihood of listing and kidney transplantation among Black individuals.^29^^,^^33^^,^^36^^,^^83^ While disparities in deceased donor kidney transplantation for Black patients were mitigated after the implementation of the Kidney Allocation System,^3^^,^^6^^,^^84^^,^^85^ racial disparities in access to live donor kidney transplantation and preemptive kidney transplantation not only persisted but worsened over time.^4^^,^^15^^,^^17^ Moreover, the COVID-19 pandemic led to significant reductions in live donor kidney transplantation due to pandemic-related policy changes and shifts in transplant center practices,^86^, ^87^, ^88^, ^89^, ^90^ which may have contributed to the observed trends in 2019–2020. Therefore, while Black patients’ barriers to deceased donor kidney transplantation were partially ameliorated due to the Kidney Allocation System, challenges in access to listing, live donor kidney transplantation, and preemptive kidney transplantation persisted; these disparities may be attributed to a combination of aforementioned individual, institutional, and structural factors.

Interestingly, we observed that Black and Hispanic patients were modestly more likely to be informed of kidney transplantation than their White counterparts, which was not seen among Asian patients. However, regardless of information status, Black and Hispanic patients were less likely to be listed and obtain a kidney transplant than their White counterparts. Interestingly, the likelihood of listing did not differ by racial and ethnic groups among patients not informed of kidney transplantation; after listing, candidates' information status was not associated with the likelihood of kidney transplantation among minoritized candidates relative to White candidates. Nonetheless, it remains uncertain whether the quality of transplant education is uniform across different racial and ethnic groups, or the criteria used to define “informed” status in CMS 2728.^91^ Existing literature suggests that variations in informed status could stem from the diverse practices of nephrologists in for-profit and non-profit centers,^92^ access to nephrology care before end-stage kidney disease diagnosis,^91^ and private insurance coverage.^91^ Further research is necessary to unravel patient-provider relationships, specifically exploring each racial and ethnic group's optimal timing and content of transplant education.^92^ Despite minoritized patients being more likely to be informed of kidney transplantation, disparities in listing and kidney transplantation persist in the subsequent steps, as highlighted above.

Our study has several strengths, including the ability to ascertain access to listing and transplantation using national data after the implementation of a major allocation policy shift, namely, the Kidney Allocation System. However, our study also has limitations. First, we relied on provider-reported information on CMS-2728 forms, which had varying quality and completeness. Specifically, the variable capturing informed status of kidney transplant options may not reflect the patient's knowledge of the transplant process. Second, we lacked granularity in race and ethnicity information to report outcomes within specific subgroups. This limitation is particularly relevant for Asian and Hispanic individuals, as these groups demonstrate heterogeneity in sociocultural, socioeconomic, and other relevant characteristics, potentially leading to varying barriers. Third, there may be potential selection bias as this analysis was based on a complete case analysis; however, since the missingness of covariates is <10%, the risk of selection bias may be low. Fourth, the study may be overpowered due to large sample sizes. Lastly, we have not discussed estimates by sex/gender due to the scope of this study.

In conclusion, our study of patients across the kidney transplant care continuum highlights that Black and Hispanic minoritized patients are slightly more likely to be informed of kidney transplantation; however, minoritized individuals face significant challenges in accessing kidney transplantation thereafter. Asian patients had better access to listing, but poorer access to transplantation. Conversely, both Black and Hispanic patients experienced significantly lower access to both listing and transplantation. These disparities likely arise from a complex interplay of systemic and individual factors acting as barriers across key stages of the kidney transplant care continuum, emphasizing the ongoing need for research and targeted interventions. Moreover, it is essential to recognize that these disparities may affect racial and ethnic groups differently. Our study underscores the urgent need for research aimed at identifying racial and ethnic-specific interventions across key stages in the kidney transplant care continuum to foster more equitable access to kidney transplantation.

## Contributors

M.A.M.D and M.N.C-C constructed the concept and design of study. M.A.M.D, Y.L, and G.M contributed to acquisition, analysis, and interpretation of data. Y.L, G.M, G.T.M, M.N.C-C, M.A.M.D, M.G.B., B.K., B.J.O., S.P.W., M.D.H., T.S.P., and D.L.S. drafted the manuscript. Y.L., G.M., G.T.M., M.N.C-C, M.A.M.D, M.G.B., B.K., B.J.O., S.P.W., M.D.H., T.S.P., and D.L.S. have revised the manuscript and provided critical input. Y.L and G.M were granted access to the data and verified it. They also contributed to statistical analysis and administrative, technical, or material support. M.A.M.D and D.L.S obtained funding. M.A.M.D, M.N.C-C, and D.L.S provided supervision for this project. Y.L., G.M., G.T.M., M.N.C-C, M.A.M.D, M.G.B., B.K., B.J.O., S.P.W., M.D.H., T.S.P., and D.L.S. have reviewed and approved the final version of this manuscript and have agreed to the decision to submit it for publication.

## Data sharing statement

The datasets used and/or analyzed during the current study are available from the United States Renal Data System (USRDS) upon Data Use Agreement (DUA) approval (https://usrds-adr.niddk.nih.gov/2023). Per the DUA between the authors and USRDS, the release of the data or the deposition of data into publicly available repositories or to individuals is not allowed.

## Disclaimer

The data reported herein have been supplied by the US Renal Data System. The interpretation and reporting of these data are the responsibility of the authors and in no way should be seen as an official policy or interpretation of the US government.

## Declaration of interests

DL Segev receives consulting fees from AstraZeneca, CareDx, Moderna Therapeutics, Novavax, Regeneron, and Springer Publishing. DL Segev also receives honoraria from AstraZeneca, CareDx, Houston Methodist, Northwell Health, Optum Health Education, Sanofi, and WebMd. DL Segev also receives payment from Springer. BJ Orandi is the associate editor for *Clinical Transplanation and Obesity*. MN Clark-Cutaia participates in the THRIVE advisory board, and is the chapter president of Sigma Theta Tau Xi (Nursing Honor Society). The remaining authors have no competing interests to declare.
