# Supplementary material for: Identifying when racial and ethnic disparities arise along the continuum of transplant care: a national registry study
Source: Lancet Reg Health Am. 2024 Oct 3;38:100895. doi: 10.1016/j.lana.2024.100895 (PMC11489072; doi:10.1016/j.lana.2024.100895)
Supplement: Supplementary Material [file mmc1.docx]

**SUPPLEMENTARY MATERIAL**

**for**

**Identifying when Racial and Ethnic Disparities Arise along the Continuum of Transplant Care: A National Registry Study**

Maya N. Clark-Cutaia PhD, ACNP-BC,* Gayathri Menon MHS,* Yiting Li MPH, Garyn T. Metoyer MD, Mary Grace Bowring MPH, Byoungjun Kim PhD, Babak J. Orandi MD, PhD, Stephen P. Wall MD, Melissa D. Hladek PhD, MSN, RN, Tanjala S. Purnell, PhD, MPH, Dorry L. Segev MD, PhD, and Mara A. McAdams-DeMarco PhD

*MNC-C and GM contributed equally to this publication

**Table of Contents**

**Supplementary Table S1.** Crude proportion of adults with end-stage kidney disease who were informed of kidney transplantation at the time dialysis initiation, stratified by race and ethnicity and year of dialysis initiation (2015-2020).

**Supplementary Table S2.** Unadjusted cumulative incidence of listing, any kidney transplantation, deceased donor kidney transplantation, live donor kidney transplantation, and preemptive kidney transplantation at 1, 3, and 5 years after dialysis initiation and listing, respectively (2015-2020).

**Supplementary Table S3.** Trends in access to listing by candidate race and ethnicity (2015-2020) (N=637 951).

**Supplementary Table S4.** Clinical and demographic characteristics of listing population (2015-2020).

**Supplementary Table S5.** Trends in access to any kidney transplantation, deceased donor kidney transplantation, live donor kidney transplantation, and preemptive kidney transplantation after listing by candidate race and ethnicity (2015-2020) (N=98 561)

**Supplementary Table S6.** Reasons for Not Informing Patients of Kidney Transplantation at Dialysis Initiation, by Race and Ethnicity among those not informed of Kidney Transplant (N=94 176)

**Supplementary Table S7.** Access to Key Stages on the Kidney Transplant Care Continuum by Race and Ethnicity and Informed Status at Dialysis Initiation (2015-2020).

**Supplementary Table S8. [Sensitivity Analysis: Fine and Gray Subdistribution Hazards Models]** Access to Key Steps on the Kidney Transplantation Care Continuum stratified by Dialysis Patients and Candidates' Race and Ethnicity (2015-2020).

**Supplementary Table S9. [Sensitivity Analysis: Medicare Beneficiaries]** Access to Key Steps on the Kidney Transplant Care Continuum among Medicare Beneficiaries, stratified by Dialysis Patients and Candidates' Race and Ethnicity (2015-2020)

**Supplementary Table S10. [Sensitivity Analysis: Time-Varying Cause-Specific Hazards Models]** Access to Key Stages on the Kidney Transplantation Care Continuum by Race and Ethnicity (2015-2020).

**Supplementary Table S11. [Sensitivity Analysis: Separate Categories for Missing Values]** Access to Key Steps in Kidney Transplantation Care Continuum stratified by Dialysis Patients and Candidates' Race and Ethnicity (2015-2020).

**Supplementary Table S12. [Sensitivity Analysis: Including Preemptively Listed Individuals]** Access to Key Stages on the Kidney Transplantation Care Continuum by Race and Ethnicity (2015-2020).

**Supplementary Table S13. [Sensitivity Analysis: Including Employment as a Covariate]** Access to Key Stages on the Kidney Transplantation Care Continuum by Race and Ethnicity (2015-2020).

**Supplementary Table S14: [Sensitivity Analysis: Separating Asian into Asian American and Native Hawaiian/Pacific Islanders]** Access to Key Stages on the Kidney Transplant Continuum of Care by Race (2015-2020).

**Supplementary Table S15. [Sensitivity Analysis: Composite outcome of Listing and Kidney Transplantation]** Access to Listing/Kidney Transplantation by Race and Ethnicity (2015-2020).

**Supplementary Figure S1.** Timeline of Kidney Transplant Care Continuum

**STROBE Checklist**

**Supplementary Table S1. Crude proportion of adults with end-stage kidney disease who were informed of kidney transplantation at the time of dialysis initiation, stratified by race and ethnicity and year of dialysis initiation (2015-2020).**

|  |  | Year of Dialysis Initiation | | |
| --- | --- | --- | --- | --- |
| Race and Ethnicity | **Informed, No. (%)** | **2015-2016** | **2017-2018** | **2019-2020** |
| Asian^a^ | 31 995 (85·4) | 9 644 (86·5) | 10 774 (86·3) | 11 577 (83·6) |
| Black^a^ | 152 725 (87·5) | 50 009 (88·9) | 50 748 (89·8) | 51 968 (84·0) |
| Hispanic | 98 133 (88·7) | 30 593 (89·6) | 32 385 (90·1) | 35 155 (86·8) |
| White^a^ | 292 740 (84·3) | 97 889 (85·0) | 101 710 (86·3) | 93 141 (81·7) |
| Total | 575 593 (85·9) | 188 135 (86·8) | 195 617 (87·8) | 191 841 (83·3) |

Crude proportions obtained from responses to Q.25 in Centers for Medicare and Medicaid Services (CMS) form 2728, which enquires whether the provider informed the individual with end-stage kidney disease of kidney transplant options at the time of dialysis initiation. CMS-2728 was revised in 2005 to include information about individuals with end-stage kidney disease being informed of transplantation options. The response to the question has the following options: Yes or No. Individuals with unknown/missing informed status (N=12 938) were excluded from crude estimates.

^a^Non-Hispanic White, Black and Asian; Asian includes Asian American, Native Hawaiian, and Pacific Islander.

**Supplementary Table S2. Unadjusted cumulative incidence of listing, any kidney transplantation, deceased donor kidney transplantation, live donor kidney transplantation, and preemptive kidney transplantation at 1, 3, and 5 years after dialysis initiation and listing, respectively (2015-2020).**

|  | Unadjusted Cumulative Incidence (%) (95% Confidence Interval) | | |
| --- | --- | --- | --- |
|  | **1 Year** | **3 Year** | **5 Year** |
| Listing^a^ |  |  |  |
| Asian | 9·6 (9·3-9·9) | 19·6 (19·1-20·1) | 23·6 (23·0-24·2) |
| Black | 6·4 (6·3-6·6) | 15·9 (15·7-16·1) | 20·5 (20·2-20·8) |
| Hispanic | 7·8 (7·6-8·0) | 18·1 (17·9-18·4) | 22·8 (22·4-23·1) |
| White | 6·5 (6·4-6·6) | 13·1 (13·0-13·3) | 16·1 (15·9-16·3) |
| Any Kidney Transplantation^b^ |  |  |  |
| Asian | 18·8 (17·9-19·7) | 38·5 (37·3-39·7) | 56·4 (54·6-58·1) |
| Black | 17·8 (17·3-18·3) | 37·7 (37·0-38·4) | 58·1 (57·1-59·2) |
| Hispanic | 21·5 (20·9-22·1) | 39·8 (39·0-40·6) | 58·1 (56·9-59·4) |
| White | 33·5 (33·0-33·9) | 58·6 (58·1-59·1) | 75·9 (75·3-76·6) |
| Deceased Donor Kidney Transplantation^b^ |  |  |  |
| Asian | 8·6 (8·0-9·3) | 23·9 (22·7-25·1) | 43·1 (41·1-45·1) |
| Black | 11·2 (10·8-11·6) | 28·2 (27·5-28·9) | 50·0 (48·8-51·2) |
| Hispanic | 10·1 (9·6-10·6) | 24·4 (23·6-25·2) | 44·8 (43·3-46·3) |
| White | 12·3 (11·9-12·6) | 32·2 (31·6-32·8) | 55·4 (54·4-56·5) |
| Live Donor Kidney Transplantation^b^ |  |  |  |
| Asian | 6·5 (5·9-7·1) | 12·6 (11·8-13·6) | 16·0 (14·7-17·3) |
| Black | 4·5 (4·2-4·7) | 8·9 (8·4-9·3) | 11·4 (10·8-12·1) |
| Hispanic | 8·3 (7·9-8·7) | 14·8 (14·1-15·4) | 18·1 (17·2-19·0) |
| White | 13·0 (12·7-13·4) | 24·1 (23·6-24·6) | 29·9 (29·1-30·7) |
| Preemptive Kidney Transplantation^b^ |  |  |  |
| Asian | 5·0 (4·5-5·5) | 7·5 (6·9-8·2) | 8·8 (7·9-9·8) |
| Black | 3·1 (2·9-3·4) | 4·8 (4·5-5·1) | 5·4 (5·0-5·8) |
| Hispanic | 4·8 (4·5-5·1) | 6·7 (6·3-7·1) | 7·4 (6·9-7·9) |
| White | 12·8 (12·5-13·1) | 19·6 (19·1-20·1) | 22·9 (22·3-23·6) |

**^a^**Excluding individuals listed prior to dialysis initiation

^b^Including individuals listed prior to dialysis initiation

**Supplementary Table S3. Trends in access to listing by candidate race and ethnicity (2015-2020) (N=637 951).**

|  |  | Adjusted Hazard Ratio (aHR) (95% Confidence Interval)^a^ | | | |
| --- | --- | --- | --- | --- | --- |
|  | **Year** | **White^b^** | **Asian^b^** | **Black^b^** | **Hispanic^b^** |
| Listing | 2015-2016 | Reference | **1·29 (1·23-1·35)** | **0·89 (0·87-0·92)** | **0·93 (0·91-0·96)** |
|  | 2017-2018 | Reference | **1·18 (1·13-1·24)** | **0·90 (0·88-0·93)** | **0·91 (0·88-0·94)** |
|  | 2019-2020 | Reference | 1·05 (0.99-1·10) | **0·80 (0·77-0·82)** | **0·72 (0·69-0·75)** |
|  | P_Trend_^c^ |  | **<0·0001** | **<0·0001** | **<0·0001** |

Bold denotes statistically significant at 2-sided α of 0·05.

^a^Adjusted HR of being listed in dialysis population (adjusted for: year of dialysis initiation, cause of end-stage kidney disease, sex, age at first diagnosis, body mass index, comorbidities [hypertension, diabetes, heart failure, atherosclerotic heart disease, peripheral vascular disease, cerebrovascular disease, chronic obstructive pulmonary disease, cancer, functional impairment, inability to transfer, alcohol use, tobacco use], and Medicare). Candidates who were listed prior to their dialysis initiation were excluded.

^b^Non-Hispanic White, Black and Asian; Asian includes Asian American, Native Hawaiian, and Pacific Islander

^c^P-value for Wald test testing the significance of the interaction between candidate race and ethnicity and year of dialysis initiation

**Supplementary Table 4. Clinical and demographic characteristics of listing population (2015-2020).**

|  | **Total** | **Asian^a^** | **Black** | **Hispanic** | **White** |
| --- | --- | --- | --- | --- | --- |
|  | N=98 561 | N=7624 | N=24 327 | N=18 448 | N=48 162 |
| **Age in years, mean(SD)** | 53·4 (13·2) | 53·1 (13·1) | 52·1 (12·6) | 50·5 (13·2) | 55·2 (13·2) |
| **Age in years, No. (%)^b^** |  |  |  |  |  |
| 18-34 | 11 028 (11·2) | 830 (10·9) | 2717 (11·2) | 2834 (15·4) | 4647 (9·6) |
| 35-49 | 24 556 (24·9) | 2035 (26·7) | 7254 (29·8) | 5247 (28·4) | 10 020 (20·8) |
| 50-64 | 42 196 (42·8) | 3210 (42·1) | 10 301 (42·3) | 7881 (42·7) | 20 804 (43·2) |
| ≥65 | 20 781 (21·1) | 1549 (20·3) | 4055 (16·7) | 2486 (13·5) | 12 691 (26·4) |
| **Female, No. (%)** | 36 378 (36·9) | 2929 (38·4) | 9955 (40·9) | 6482 (35·1) | 17 012 (35·3) |
| **Primary cause of end-stage kidney disease, No. (%)** |  |  |  |  |  |
| Diabetes | 36 089 (36·6) | 3151 (41·3) | 8787 (36·1) | 8659 (46·9) | 15 492 (32·2) |
| Hypertension | 25 949 (26·3) | 1811 (23·8) | 9659 (39·7) | 4194 (22·7) | 10 285 (21·4) |
| Glomerulonephritis | 16 592 (16·8) | 1644 (21·6) | 3303 (13·6) | 2912 (15·8) | 8733 (18·1) |
| Others | 19 931 (20·2) | 1018 (13·4) | 2578 (10·6) | 2683 (14·5) | 13 652 (28·3) |
| **BMI in kg/m^2^, median [IQR]** | 28·8 [25·0-33·2] | 25·9 [22·7-29·9] | 29·9 [25·8-34·4] | 28·2 [24·8-32·3] | 29·0 [25·1-33·5] |
| **BMI in kg/m^2^, No. (%)** |  |  |  |  |  |
| 14-17·9 | 1126 (1·1) | 167 (2·2) | 223 (0·9) | 199 (1·1) | 537 (1·1) |
| 18-24·9 | 23 774 (24·1) | 3093 (40·6) | 4756 (19·6) | 4721 (25·6) | 11 204 (23·3) |
| 25-29·9 | 31 401 (31·9) | 2496 (32·7) | 7254 (29·8) | 6482 (35·1) | 15 169 (31·5) |
| ≥30 | 42 260 (42·9) | 1868 (24·5) | 12 094 (49·7) | 7046 (38·2) | 21 252 (44·1) |
| **Calculated PRA>80%, No. (%)** | 6820 (6·9) | 467 (6·1) | 2360 (9·7) | 1243 (6·7) | 2750 (5·7) |
| **Comorbidities, No. (%)** |  |  |  |  |  |
| Diabetes | 44 006 (44·6) | 3614 (47·4) | 11 557 (47·5) | 9559 (51·8) | 19 276 (40·0) |
| Hypertension | 86 305 (87·6) | 6723 (88·2) | 22 453 (92·3) | 16 174 (87·7) | 40 955 (85·0) |
| Alcohol Dependence | 1307 (1·3) | 28 (0·4) | 179 (0·7) | 233 (1·3) | 867 (1·8) |
| Cancer | 3059 (3·1) | 119 (1·6) | 583 (2·4) | 265 (1·4) | 2092 (4·3) |
| Atherosclerotic Heart Disease | 5711 (5·8) | 466 (6·1) | 1108 (4·6) | 910 (4·9) | 3227 (6·7) |
| Cerebrovascular Disease | 3390 (3·4) | 218 (2·9) | 1069 (4·4) | 513 (2·8) | 1590 (3·3) |
| Heart failure | 10 465 (10·6) | 695 (9·1) | 3435 (14·1) | 1815 (9·8) | 4520 (9·4) |
| Inability to Transfer | 280 (0·3) | 16 (0·2) | 70 (0·3) | 53 (0·3) | 141 (0·3) |
| Tobacco use | 3029 (3·1) | 136 (1·8) | 894 (3·7) | 269 (1·5) | 1730 (3·6) |
| Peripheral Vascular Disease | 3388 (3·4) | 174 (2·3) | 766 (3·1) | 702 (3·8) | 1746 (3·6) |
| Chronic Obstructive Pulmonary Disease | 1931 (2·0) | 97 (1·3) | 478 (2·0) | 181 (1·0) | 1175 (2·4) |
| Functional Impairment | 3283 (3·3) | 232 (3·0) | 775 (3·2) | 756 (4·1) | 1520 (3·2) |
| **Medicare, No. (%)^b^** | 33 221 (33·7) | 1 854 (24·3) | 7799 (32·1) | 4701 (25·5) | 18 867 (39·2) |
| **Employment Status, No. (%)^b^** |  |  |  |  |  |
| Employed | 34 668 (35·2) | 2925 (38·4) | 8164 (33·6) | 5448 (29·5) | 18 131 (37·6) |
| Unemployed | 23 633 (24·0) | 1958 (25·7) | 6807 (28·0) | 6460 (35·0) | 8408 (17·5) |
| Retired | 31 975 (32·4) | 1980 (26·0) | 7321 (30·1) | 4856 (26·3) | 17 818 (37·0) |
| Other | 8285 (8·4) | 761 (10·0) | 2035 (8·4) | 1684 (9·1) | 3805 (7·9) |
| **Nephrology Care Prior to End-Stage Kidney Disease Diagnosis, No. (%)** |  |  |  |  |  |
| Yes | 74 571 (75·7) | 5 832 (76·5) | 17 506 (72·0) | 12 486 (67·7) | 38 747 (80·5) |
| No | 13 787 (14·0) | 1047 (13·7) | 3935 (16·2) | 3615 (19·6) | 5190 (10·8) |
| Unknown | 745 (9·8) | 2886 (11·9) | 2347 (12·7) | 4225 (8·8) | 745 (9·8) |

SD = standard deviation; BMI: body mass index; IQR: interquartile range; PRA= panel reactive antibody

^a^Non-Hispanic White, Black, and Asian; Asian includes Asian American, Native Hawaiian, and Pacific Islander

^b^At dialysis initiation

**Supplementary Table 5. Trends in access to any kidney transplantation, deceased donor kidney transplantation, live donor kidney transplantation, and preemptive kidney transplantation after listing by candidate race and ethnicity (2015-2020) (N=98 561)**

|  |  | Adjusted Hazard Ratio (aHR) (95% Confidence Interval)^a^ | | | |
| --- | --- | --- | --- | --- | --- |
|  | **Year^c^** | **White^b^** | **Asian^b^** | **Black^b^** | **Hispanic^b^** |
| Any Kidney Transplantation^d^ | 2015-2016 | Reference | **0·59 (0·55-0·63)** | **0·60 (0·57-0·63)** | **0·68 (0·64-0·71)** |
|  | 2017-2018 | Reference | **0·55 (0·52-0·59)** | **0·62 (0·60-0·64)** | **0·63 (0·61-0·66)** |
|  | 2019-2020 | Reference | **0·53 (0·49-0·57)** | **0·61 (0·59-0·64)** | **0·63 (0·60-0·66)** |
|  | **P_Trend_**^c^ |  | 0·08 | 0·51 | **0·05** |
| Deceased Donor Kidney transplantation^d^ | 2015-2016 | Reference | **0·79 (0·72-0·86)** | **0·90 (0·85-0·96)** | **0·83 (0·77-0·89)** |
|  | 2017-2018 | Reference | **0·69 (0·64-0·75)** | **0·93 (0·88-0·97)** | **0·79 (0·75-0·84)** |
|  | 2019-2020 | Reference | **0·61 (0·56-0·67)** | **0·91 (0·87-0·96)** | **0·77 (0·72-0·82)** |
|  | **P_Trend_**^c^ |  | **0·0007** | 0·81 | 0·29 |
| Live Donor Kidney Transplantation^d^ | 2015-2016 | Reference | **0·45 (0·40-0·52)** | **0·39 (0·35-0·43)** | **0·62 (0·57-0·68)** |
|  | 2017-2018 | Reference | **0·42 (0·38-0·48)** | **0·35 (0·32-0·38)** | **0·53 (0·49-0·57)** |
|  | 2019-2020 | Reference | **0·43 (0·38-0·49)** | **0·31 (0·28-0·34)** | **0·53 (0·49-0·58)** |
|  | **P_Trend_**^c^ |  | 0·71 | **0·003** | **0·009** |
| Preemptive Kidney Transplantation^d^ | 2015-2016 | Reference | **0·49 (0·42-0·57)** | **0·39 (0·35-0·44)** | **0·57 (0·52-0·64)** |
|  | 2017-2018 | Reference | **0·45 (0·39-0·53)** | **0·34 (0·31-0·38)** | **0·45 (0·40-0·50)** |
|  | 2019-2020 | Reference | **0·44 (0·37-0·53)** | **0·29 (0·25-0·33)** | **0·39 (0·34-0·44)** |
|  | **P_Trend_**^c^ |  | 0·64 | **0·004** | **<0·0001** |

^a^Adjusted HR of being transplanted in listing population (adjusted for: cause of end-stage kidney disease, sex, age at listing, body mass index, hypertension, diabetes, heart failure, atherosclerotic heart disease, peripheral vascular disease, cerebrovascular disease, chronic obstructive pulmonary disease, cancer, functional impairment, inability to transfer, alcohol use, tobacco use, blood group, calculated panel reactive antibody, and Medicare).

^b^Non-Hispanic White, Black, and Asian; Asian includes Asian American, Native Hawaiian, and Pacific Islander

^c^P-value for Wald test testing the significance of the interaction between candidate race and ethnicity and year of dialysis initiation

^d^Includes individuals who were listed prior to dialysis initiation

**Supplementary Table 6. Reasons for Not Informing Patients of Kidney Transplantation at Dialysis Initiation, by Race and Ethnicity among those not informed of Kidney Transplant (N=94 176)**

|  |  | Race and Ethnicity, No. (Col % ) | | | |
| --- | --- | --- | --- | --- | --- |
| Reasons for Not Informing | **Not Informed, No. (%)** | **Asian^a^** | **Black** | **Hispanic** | **White** |
| Medically Unfit | 25 391 (27.0) | 1405 (25.6) | 5027 (23·0) | 2583 (20·7) | 16 376 (30.1) |
| Unsuitable Age | 17 481 (18.6) | 1209 (22.1) | 3023 (13.8) | 1854 (14.9) | 11 395 (21.0) |
| Psychologically Unfit | 2111 (2.2) | 103 (1.9) | 687 (3.1) | 246 (2.0) | 1075 (2.0) |
| Patient Declined | 1424 (1.5) | 71 (1.3) | 243 (1.1) | 169 (1.4) | 941 (1.7) |
| Other Reasons | 5790 (6.2) | 780 (14.2) | 1308 (6.0) | 1147 (9.2) | 2555 (4.7) |
| Patient Unassessed | 50 119 (46.8) | 2435 (44.3) | 13 102 (59.9) | 7578 (60.8) | 27 004 (49·7) |
| No reason provided | 8 (0·1) | 0 (0·0) | 2 (0·01) | 1 (0·01) | 5 (0.01) |
| Mean number of reasons for not informing (SD) | - | 1.1 (0.3) | 1.1 (0.3) | 1.1 (0.3) | 1.1 (0.3) |

Reasons for not informing are not mutually exclusive. The column percentages may not be equal to 100%, since an individual may have >1 reason they’re not informed of kidney transplant options.

^a^Asian includes Asian American, Native Hawaiian, and Pacific Islander

**Supplementary Table 7. Access to Key Stages on the Kidney Transplant Care Continuum by Race and Ethnicity and Informed Status at Dialysis Initiation (2015-2020).**

|  | Adjusted Hazard Ratio (aHR) (95% Confidence Interval)^a^ | | | |
| --- | --- | --- | --- | --- |
|  | **White^b^** | **Asian^b^** | **Black^b^** | **Hispanic** |
| Listed (N=637 868) |  |  |  |  |
| Not Informed | Reference | 1.01 (0.90-1.13) | 1.06 (0.99-1.14) | 1.00 (0.93-1.08) |
| Informed | Reference | **1.21 (1.17-1.24)** | **0.85 (0.84-0.87)** | **0.85 (0.84-0.87)** |
| P-value for interaction |  | **0.002** | **<0·0001** | **<0·0001** |
| Transplanted^c^ (N=87 984) |  |  |  |  |
| Any Kidney Transplant |  |  |  |  |
| Not Informed | Reference | **0.63 (0.52-0.77)** | **0.68 (0.60-0.77)** | **0.66 (0.57-0.65)** |
| Informed | Reference | **0.56 (0.53-0.58)** | **0.65 (0.63-0.67)** | **0.66 (0.64-0.68)** |
| P-value for interaction |  | 0.23 | 0.41 | 0.97 |
| Deceased Donor Kidney Transplant |  |  |  |  |
| Not Informed | Reference | **0.79 (0.62-0.99)** | 0.91 (0.79-1.05) | **0.80 (0.68-0.94)** |
| Informed | Reference | **0.66 (0.63-0.70)** | **0.88 (0.85-0.91)** | **0.75 (0.72-0.77)** |
| P-value for interaction |  | 0.15 | 0.64 | 0.38 |
| Live Door Kidney Transplant |  |  |  |  |
| Not Informed | Reference | **0.39 (0.26-0.58)** | **0.31 (0.24-0.41)** | **0.43 (0.34-0.56)** |
| Informed | Reference | **0.42 (0.39-0.45)** | **0.33 (0.31-0.35)** | **0.54 (0.51-0.56)** |
| P-value for interaction |  | 0.76 | 0.76 | 0.11 |

Bold denotes statistically significant at a two-sided p-value of 0·05. Results for preemptive kidney transplant are suppressed due to insufficient power, as >99% of preemptive kidney transplant recipients were missing information status. Individuals with missing informed status were excluded from the analysis.

^a^Adjusted HR of being listed in the dialysis population (adjusted for: year of dialysis initiation, cause of end-stage kidney disease (diabetes, hypertension, glomerulonephritis, and other), sex, age at dialysis initiation, body mass index (BMI), comorbidities (hypertension, diabetes, heart failure, atherosclerotic heart disease, peripheral vascular disease, cerebrovascular disease, chronic obstructive pulmonary disease, cancer, functional impairment, inability to transfer, alcohol use, and tobacco use), and Medicare at dialysis initiation). Adjusted HR of being transplanted after listing (adjusted for: year of listing, cause of end-stage kidney disease, sex, age at listing, BMI, comorbidities, blood group, calculated panel reactive antibody, and Medicare at dialysis initiation).

^b^Non-Hispanic White, Black, and Asian; Asian includes Asian American, Native Hawaiian, and Pacific Islander.

^c^Includes individuals who were listed prior to dialysis initiation.

**Supplementary Table S8. [Sensitivity Analysis: Fine and Gray Subdistribution Hazards Models] Access to Key Steps on the Kidney Transplant Care Continuum stratified by Dialysis Patients and Candidates' Race and Ethnicity (2015-2020).**

|  | Adjusted Sub-Hazard Ratio (aSHR) (95% Confidence Interval)^a^ | | | |
| --- | --- | --- | --- | --- |
| Outcome | **White^b^** | **Asian^b^** | **Black^b^** | **Hispanic** |
| Listed (N=637 951) | Reference | **1·29 (1·25-1·32)** | **0·92 (0·90-0·93)** | **0·94 (0·92-0·96)** |
| Transplanted^c^ (N=98 561) |  |  |  |  |
| Any Kidney Transplant | Reference | **0·57 (0·55-0·60)** | **0·63 (0·61-0·64)** | **0·66 (0·64-0·68)** |
| Deceased Donor Kidney Transplant | Reference | **0·73 (0·69-0·77)** | **0·96 (0·93-0·99)** | **0·82 (0·79-0·85)** |
| Live Donor Kidney Transplant | Reference | **0·44 (0·41-0·47)** | **0·35 (0·33-0·37)** | **0·56 (0·53-0·59)** |
| Preemptive Kidney Transplant | Reference | **0·47 (0·43-0·51)** | **0·35 (0·32-0·37)** | **0·47 (0·44-0·51)** |

Bold denotes statistically significant at a two-sided p-value of 0·05.

^a^Adjusted SHR of being listed in dialysis population (adjusted for: year of dialysis initiation, cause of end-stage kidney disease (diabetes, hypertension, glomerulonephritis, and other), sex, age at dialysis initiation, body mass index (BMI), comorbidities (hypertension, diabetes, heart failure, atherosclerotic heart disease, peripheral vascular disease, cerebrovascular disease, chronic obstructive pulmonary disease, cancer, functional impairment, inability to transfer, alcohol use, and tobacco use), and Medicare at dialysis initiation), treating death and kidney transplant as competing risks. Adjusted SHR of being transplanted in listing population (adjusted for: year of listing, cause of end-stage kidney disease, sex, age at listing, BMI, comorbidities, blood group, calculated panel reactive antibody, and Medicare at dialysis initiation), treating death as competing risk.

^b^Non-Hispanic White, Black, and Asian; Asian includes Asian American, Native Hawaiian, and Pacific Islander.

^c^Includes individuals who were listed prior to dialysis initiation.

**Supplementary Table S9. [Sensitivity Analysis: Medicare Beneficiaries] Access to Key Steps on the Kidney Transplant Care Continuum among Medicare Beneficiaries, stratified by Dialysis Patients and Candidates' Race and Ethnicity (2015-2020)**

|  | Adjusted Hazard Ratio (aHR) (95% Confidence Interval)^a^ | | | |
| --- | --- | --- | --- | --- |
| Outcome | **White^b^** | **Asian^b^** | **Black^b^** | **Hispanic** |
| Listed (N=402 212) | Reference | **1·26 (1·18-1·33)** | 1·00 (0·97-1·03) | **1·12 (1·08-1·16)** |
|  |  |  |  |  |
| Transplanted^c^ (N=33 221) |  |  |  |  |
| Any Kidney Transplant | Reference | **0·62 (0·57-0·67)** | **0·65 (0·63-0·68)** | **0·73 (0·70-0·77)** |
| Deceased Donor Kidney Transplant | Reference | **0·71 (0·64-0·79)** | 0·95 (0·90-1·00) | **0·89 (0·83-0·96)** |
| Live Donor Kidney Transplant | Reference | **0·49 (0·41-0·58)** | **0·36 (0·32-0·40)** | **0·66 (0·58-0·74)** |
| Preemptive Kidney Transplant | Reference | **0·56 (0·48-0·66)** | **0·37 (0·33-0·41)** | **0·52 (0·46-0·59)** |

Bold denotes statistically significant at a two-sided p-value of 0·05. Medicare status is from the time of dialysis initiation.

^a^Adjusted HR of being listed in the dialysis population (adjusted for: year of dialysis initiation, cause of end-stage kidney disease (diabetes, hypertension, glomerulonephritis, and other), sex, age at dialysis initiation, body mass index [BMI], and comorbidities [hypertension, diabetes, heart failure, atherosclerotic heart disease, peripheral vascular disease, cerebrovascular disease, chronic obstructive pulmonary disease, cancer, functional impairment, inability to transfer, alcohol use, tobacco use]). Adjusted HR of being transplanted after listing (adjusted for: year of listing, cause of end-stage kidney disease, sex, age at listing, BMI, comorbidities, blood group, and calculated panel reactive antibody).

^b^Non-Hispanic White, Black, and Asian; Asian includes Asian American, Native Hawaiian, and Pacific Islander

^c^Includes individuals who were listed prior to dialysis initiation

**Supplementary Table S10. [Sensitivity Analysis: Time-Varying Cause-Specific Hazards Models] Access to Key Stages on the Kidney Transplant Care Continuum by Race and Ethnicity (2015-2020).**

|  | Adjusted Hazard Ratio (aHR) (95% Confidence Interval)^a^ | | | |
| --- | --- | --- | --- | --- |
|  | **White^b^** | **Asian^b^** | **Black^b^** | **Hispanic** |
| Listed (N=637 951)  First Year | Reference | **1·09 (1·04-1·14)** | **0·66 (0·64-0·68)** | **0·68 (0·66-0·70)** |
| Change per year for each subsequent year | Reference | **1·07 (1·04-1·10)** | **1·23 (1·21-1·25)** | **1·20 (1·18-1·22)** |
|  |  |  |  |  |
| Transplanted^c^ (N=98 561) |  |  |  |  |
| Any Kidney Transplantation |  |  |  |  |
| First Year | Reference | **0·50 (0·48-0·53)** | **0·52 (0·50-0·54)** | **0·65 (0·62-0·67)** |
| Change per year for each subsequent year | Reference | **1·07 (1·05-1·10)** | **1·11 (1·10-1·13)** | 1·00 (0·98-1·02) |
| Deceased Donor Kidney Transplantation |  |  |  |  |
| First Year | Reference | **0·66 (0·61-0·72)** | **0·91 (0·87-0·96)** | **0·85 (0·80-0·90)** |
| Change per year for each subsequent year | Reference | 1·02 (0·99-1·05) | 1·00 (0·98-1·02) | **0·96 (0·94-0·99)** |
| Live Donor Kidney Transplantation |  |  |  |  |
| First Year | Reference | **0·42 (0·38-0·47)** | **0·34 (0·31-0·37)** | **0·63 (0·59-0·67)** |
| Change per year for each subsequent year | Reference | 1·02 (0·96-1·09) | 1·01 (0·96-1·06) | **0·89 (0·85-0·93)** |
| Preemptive Kidney Transplantation |  |  |  |  |
| First Year | Reference | **0·50 (0·44-0·56)** | **0·40 (0·37-0·44)** | **0·59 (0·54-0·65)** |
| Change per year for each subsequent year | Reference | 0·92 (0·83-1·02) | **0·83 (0·76-0·90)** | **0·73 (0·67-0·80)** |

Bold denotes statistically significant at a two-sided p-value of 0·05.

^a^Adjusted HR of being listed in the dialysis population (adjusted for: year of dialysis initiation, cause of end-stage kidney disease (diabetes, hypertension, glomerulonephritis, and other), sex, age at dialysis initiation, body mass index (BMI), comorbidities [hypertension, diabetes, heart failure, atherosclerotic heart disease, peripheral vascular disease, cerebrovascular disease, chronic obstructive pulmonary disease, cancer, functional impairment, inability to transfer, alcohol use, and tobacco use], and Medicare at dialysis initiation). Adjusted HR of being transplanted after listing (adjusted for: year of listing, cause of end-stage kidney disease, sex, age at listing, BMI, comorbidities, blood group, calculated panel reactive antibody, and Medicare at dialysis initiation). Models included an interaction between race and ethnicity and time to model time-varying hazard ratios (for e.g., the hazard ratio of listing, which was 11% higher among Asian patients relative to White patients, increased by 7% each subsequent year relative to White patients).

^b^Non-Hispanic White, Black and Asian; Asian includes Asian American, Native Hawaiian, and Pacific Islander

^c^Includes individuals who were listed prior to dialysis initiation

**Supplementary Table S11. [Sensitivity Analysis: Separate Categories for Missing Values] Access to Key Steps on Kidney Transplant Care Continuum, stratified by Dialysis Patients and Candidates' Race and Ethnicity (2015-2020).**

|  | Adjusted Hazard Ratio (aHR) (95% Confidence Interval)^a^ | | | |
| --- | --- | --- | --- | --- |
| Outcome | **White^b^** | **Asian^b^** | **Black^b^** | **Hispanic** |
| Listed (N=699 300) | Reference | **1·16 (1·13-1·20)** | **0·87 (0·86-0·88)** | **0·87 (0·85-0·89)** |
|  |  |  |  |  |
| Transplanted^c^ (N= 113 267) |  |  |  |  |
| Any Kidney Transplant | Reference | **0·57 (0·55-0·59)** | **0·63 (0·62-0·64)** | **0·65 (0·64-0·67)** |
| Deceased Donor Kidney Transplant | Reference | **0·71 (0·68-0·75)** | **0·95 (0·92-0·98)** | **0·82 (0·79-0·85)** |
| Live Donor Kidney Transplant | Reference | **0·46 (0·43-0·49)** | **0·34 (0·33-0·36)** | **0·56 (0·54-0·59)** |
| Preemptive Kidney Transplant | Reference | **0·46 (0·41-0·50)** | **0·34 (0·33-0·36)** | **0·46 (0·43-0·49)** |

Bold denotes statistically significant at a two-sided p-value of 0·05. Missing values of categorical variables (cause of end-stage kidney disease, body mass index [BMI], blood type [for listing population only]) and binary variables (comorbidities: hypertension, diabetes, heart failure, atherosclerotic heart disease, peripheral vascular disease, cerebrovascular disease, chronic obstructive pulmonary disease, cancer, functional impairment, inability to transfer, alcohol use, tobacco use) were given a separate category.

^a^Adjusted HR of being listed in the dialysis population (adjusted for: year of dialysis initiation, cause of end-stage kidney disease (diabetes, hypertension, glomerulonephritis, and other), sex, age at dialysis initiation, BMI, comorbidities, and Medicare at dialysis initiation). Adjusted HR of being transplanted after listing (adjusted for: year of listing, cause of end-stage kidney disease, sex, age at listing, BMI, comorbidities, blood group, calculated panel reactive antibody, and Medicare at dialysis initiation).

^b^Non-Hispanic White, Black, and Asian; Asian includes Asian American, Native Hawaiian, and Pacific Islander.

^c^Includes individuals who were listed prior to dialysis initiation.

**Supplementary Table S12. [Sensitivity Analysis: Including Preemptively Listed Individuals] Access to Key Stages on the Kidney Transplant Care Continuum by Race and Ethnicity (2015-2020).**

|  | Adjusted Hazard Ratio (aHR) (95% Confidence Interval)^a^ | | | |
| --- | --- | --- | --- | --- |
|  | **White^b^** | **Asian^b^** | **Black^b^** | **Hispanic** |
| Listed (N=682 707) | Reference | **1·21 (1·18-1·24)** | **0·81 (0.80-0·83)** | **0·81 (0·79-0·82)** |
|  |  |  |  |  |
| Transplanted (N=98 561) |  |  |  |  |
| Any Kidney Transplant | Reference | **0·56 (0·54-0·58)** | **0·61 (0·60-0·63)** | **0·64 (0·63-0·66)** |
| Deceased Donor Kidney Transplant | Reference | **0·69 (0·66-0·73)** | **0·92 (0·89-0·94)** | **0·79 (0·76-0·82)** |
| Live Donor Kidney Transplant | Reference | **0·43 (0·40-0·47)** | **0·34 (0·33-0·36)** | **0·55 (0·53-0·58)** |
| Preemptive Kidney Transplant | Reference | **0·46 (0·42-0·51)** | **0·35 (0·32-0·37)** | **0·47 (0·44-0·51)** |

Bold denotes statistically significant at a two-sided p-value of 0·05. Individuals who were preemptively listed (i.e. listed before dialysis initiation) were included in the dialysis population by assigning them ½ day at risk.

^a^Adjusted HR of being listed in the dialysis population (adjusted for: year of dialysis initiation, cause of end-stage kidney disease (diabetes, hypertension, glomerulonephritis, and other), sex, age at dialysis initiation, body mass index [BMI], comorbidities [hypertension, diabetes, heart failure, atherosclerotic heart disease, peripheral vascular disease, cerebrovascular disease, chronic obstructive pulmonary disease, cancer, functional impairment, inability to transfer, alcohol use, and tobacco use], and Medicare at dialysis initiation). Adjusted HR of being transplanted after listing (adjusted for: year of listing, cause of end-stage kidney disease, sex, age at listing, BMI, comorbidities, blood group, calculated panel reactive antibody, and Medicare at dialysis initiation).

^b^Non-Hispanic White, Black, and Asian; Asian includes Asian American, Native Hawaiian, and Pacific Islander

**Supplementary Table S13. [Sensitivity Analysis: Including Employment as a Covariate] Access to Key Stages on the Kidney Transplant Care Continuum by Race and Ethnicity (2015-2020).**

|  | Adjusted Hazard Ratio (aHR) (95% Confidence Interval)^a^ | | | |
| --- | --- | --- | --- | --- |
|  | **White^b^** | **Asian^b^** | **Black^b^** | **Hispanic** |
| Listed (N=637 951) | Reference | **1·15 (1·12-1·18)** | **0·89 (0·87-0·91)** | **0·91 (0·89-0·93)** |
|  |  |  |  |  |
| Transplanted (N=98 561)^c^ |  |  |  |  |
| Any Kidney Transplant | Reference | **0·56 (0·54-0·58)** | **0·62 (0·60-0·63)** | **0·66 (0·65-0·68)** |
| Deceased Donor Kidney Transplant | Reference | **0·69 (0·66-0·73)** | **0·91 (0·88-0·94)** | **0·79 (0·76-0·82)** |
| Live Donor Kidney Transplant | Reference | **0·44 (0·41-0·47)** | **0·35 (0·33-0·37)** | **0·58 (0·56-0·61)** |
| Preemptive Kidney Transplant | Reference | **0·48 (0·44-0·53)** | **0·36 (0·34-0·39)** | **0·52 (0·49-0·56)** |

Bold denotes statistically significant at a two-sided p-value of 0·05.

^a^Adjusted HR of being listed in the dialysis population (adjusted for: year of dialysis initiation, cause of end-stage kidney disease (diabetes, hypertension, glomerulonephritis, and other), sex, age at dialysis initiation, body mass index [BMI], comorbidities [hypertension, diabetes, heart failure, atherosclerotic heart disease, peripheral vascular disease, cerebrovascular disease, chronic obstructive pulmonary disease, cancer, functional impairment, inability to transfer, alcohol use, and tobacco use], employment, and Medicare at dialysis initiation). Adjusted HR of being transplanted after listing (adjusted for: year of listing, cause of end-stage kidney disease, sex, age at listing, BMI, comorbidities, blood group, calculated panel reactive antibody, employment, and Medicare at dialysis initiation).

^b^Non-Hispanic White, Black, and Asian; Asian includes Asian American, Native Hawaiian, and Pacific Islander

^c^Includes individuals who were listed prior to dialysis initiation

**Supplementary Table S14. [Sensitivity Analysis: Separating Asian into Asian American and Native Hawaiian/Pacific Islanders] Access to Key Stages on the Kidney Transplant Care Continuum by Race (2015-2020).**

|  | Adjusted Hazard Ratio (aHR) (95% Confidence Interval)^a^ | | |
| --- | --- | --- | --- |
|  | White^b^ | Asian American^b^ | Native Hawaiian/Pacific Islander^b^ |
| Listed (N=364 204) | Reference | **1.30 (1.26-1.34)** | **0.72 (0.68-0.77)** |
| N | 356 656 | 30 551 | 7624 |
| Transplanted^c^ (N=55 786) |  |  |  |
| Any Kidney Transplant | Reference | **0.56 (0.54-0.58)** | **0.54 (0.48-0.59)** |
| Deceased Donor Kidney Transplant | Reference | **0.68 (0.65-0.72)** | **0.75 (0.66-0.85)** |
| Live Donor Kidney Transplant | Reference | **0.44 (0.41-0.48)** | **0.37 (0.30-0.45)** |
| Preemptive Kidney Transplant | Reference | **0.47 (0.53-0.52)** | **0.27 (0.20-0.37)** |
| N | 48 162 | 6547 | 1077 |

Bold denotes statistically significant at a two-sided p-value of 0·05.

^a^Adjusted HR of being listed in the dialysis population (adjusted for: year of dialysis initiation, cause of end-stage kidney disease (diabetes, hypertension, glomerulonephritis, and other), sex, age at dialysis initiation, body mass index [BMI], comorbidities [hypertension, diabetes, heart failure, atherosclerotic heart disease, peripheral vascular disease, cerebrovascular disease, chronic obstructive pulmonary disease, cancer, functional impairment, inability to transfer, alcohol use, and tobacco use], and Medicare at dialysis initiation). Adjusted HR of being transplanted after listing (adjusted for: year of listing, cause of end-stage kidney disease, sex, age at listing, BMI, comorbidities, blood group, calculated panel reactive antibody, and Medicare at dialysis initiation).

^b^Non-Hispanic

^c^Includes individuals who were listed prior to dialysis initiation.

**Supplementary Table S15. [Sensitivity Analysis: Composite outcome of Listing and Kidney Transplantation] Access to Listing/Kidney Transplantation by Race and Ethnicity (2015-2020).**

|  | Adjusted Hazard Ratio (aHR) (95% Confidence Interval)^a^ | | | |
| --- | --- | --- | --- | --- |
|  | **White^b^** | **Asian^b^** | **Black^b^** | **Hispanic** |
| Listing or Kidney Transplantation (N=637 951) | Reference | **1.17 (1.14-1.21)** | **0.87 (0.85-0.88)** | **0.86 (0.84-0.88)** |

Bold denotes statistically significant at a two-sided p-value of 0·05.

^a^Adjusted HR of being listed in the dialysis population (adjusted for: year of dialysis initiation, cause of end-stage kidney disease (diabetes, hypertension, glomerulonephritis, and other), sex, age at dialysis initiation, body mass index [BMI], comorbidities [hypertension, diabetes, heart failure, atherosclerotic heart disease, peripheral vascular disease, cerebrovascular disease, chronic obstructive pulmonary disease, cancer, functional impairment, inability to transfer, alcohol use, and tobacco use], and Medicare at dialysis initiation).

^b^Non-Hispanic; Asian includes Asian American, Native Hawaiian, and Pacific Islander

**Supplementary Figure S1. Timeline of Kidney Transplant Care Continuum**


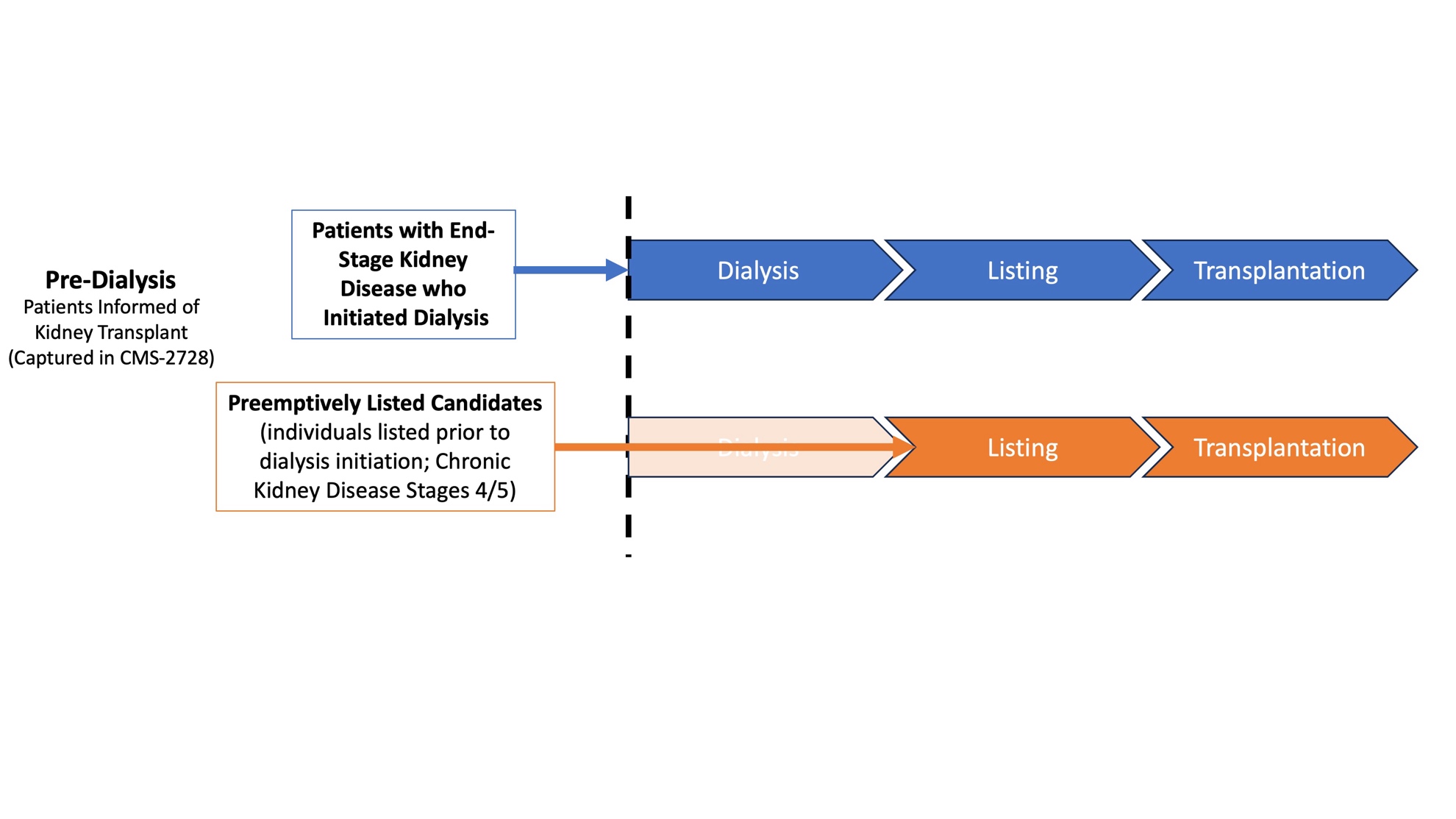


**STROBE Statement**—Checklist of items that should be included in reports of ***cohort studies***

|  | **Item No** | **Recommendation** | **Page No** |
| --- | --- | --- | --- |
| **Title and abstract** | 1 | (*a*) Indicate the study’s design with a commonly used term in the title or the abstract | 1 |
|  |  | (*b*) Provide in the abstract an informative and balanced summary of what was done and what was found | 4-5 |
| **Introduction** | | | |
| Background/rationale | 2 | Explain the scientific background and rationale for the investigation being reported | 8-9 |
| Objectives | 3 | State specific objectives, including any prespecified hypotheses | 9 |
| **Methods** | | | |
| Study design | 4 | Present key elements of study design early in the paper | 10-11 |
| Setting | 5 | Describe the setting, locations, and relevant dates, including periods of recruitment, exposure, follow-up, and data collection | 10-11 |
| Participants | 6 | (*a*) Give the eligibility criteria, and the sources and methods of selection of participants. Describe methods of follow-up | 10-11 |
|  |  | (*b*) For matched studies, give matching criteria and number of exposed and unexposed | - |
| Variables | 7 | Clearly define all outcomes, exposures, predictors, potential confounders, and effect modifiers. Give diagnostic criteria, if applicable | 11-14 |
| Data sources/ measurement | 8* | For each variable of interest, give sources of data and details of methods of assessment (measurement). Describe comparability of assessment methods if there is more than one group | 11-12 |
| Bias | 9 | Describe any efforts to address potential sources of bias | 15, 24 |
| Study size | 10 | Explain how the study size was arrived at | 10 |
| Quantitative variables | 11 | Explain how quantitative variables were handled in the analyses. If applicable, describe which groupings were chosen and why | 11-12 |
| Statistical methods | 12 | (*a*) Describe all statistical methods, including those used to control for confounding | 12-15 |
|  |  | (*b*) Describe any methods used to examine subgroups and interactions | 12-13 |
|  |  | (*c*) Explain how missing data were addressed | 13 |
|  |  | (*d*) If applicable, explain how loss to follow-up was addressed | 13-14 |
|  |  | (*e*) Describe any sensitivity analyses | 14-15 |
| **Results** | | |  |
| Participants | 13* | (a) Report numbers of individuals at each stage of study—eg numbers potentially eligible, examined for eligibility, confirmed eligible, included in the study, completing follow-up, and analysed | 10 (Fig 1, Supp Fig S2) |
|  |  | (b) Give reasons for non-participation at each stage | Fig 1, Supp Fig S2 |
|  |  | (c) Consider use of a flow diagram | Fig 1, Supp Fig S2 |
| Descriptive data | 14* | (a) Give characteristics of study participants (eg demographic, clinical, social) and information on exposures and potential confounders | 15, 17 |
|  |  | (b) Indicate number of participants with missing data for each variable of interest | Figure 1 |
|  |  | (c) Summarise follow-up time (eg, average and total amount) | 16, 18 |
| Outcome data | 15* | Report numbers of outcome events or summary measures over time | Table 3 |

| Main results | 16 | (*a*) Give unadjusted estimates and, if applicable, confounder-adjusted estimates and their precision (eg, 95% confidence interval). Make clear which confounders were adjusted for and why they were included | 15-18 |
| --- | --- | --- | --- |
|  |  | (*b*) Report category boundaries when continuous variables were categorized | 12 |
|  |  | (*c*) If relevant, consider translating estimates of relative risk into absolute risk for a meaningful time period | 15-18 |
| Other analyses | 17 | Report other analyses done—eg analyses of subgroups and interactions, and sensitivity analyses | 19 |
| **Discussion** | | | |
| Key results | 18 | Summarise key results with reference to study objectives | 20 |
| Limitations | 19 | Discuss limitations of the study, taking into account sources of potential bias or imprecision. Discuss both direction and magnitude of any potential bias | 23-24 |
| Interpretation | 20 | Give a cautious overall interpretation of results considering objectives, limitations, multiplicity of analyses, results from similar studies, and other relevant evidence | 21-23 |
| Generalisability | 21 | Discuss the generalisability (external validity) of the study results | 21-24 |
| **Other information** | | | |
| Funding | 22 | Give the source of funding and the role of the funders for the present study and, if applicable, for the original study on which the present article is based | 26-27 |
